# Supplementary material for: Inhibition of Dipeptidyl Peptidase-4 Activates Autophagy to Promote Survival of Breast Cancer Cells via the mTOR/HIF-1α Pathway
Source: Cancers (Basel). 2023 Sep 12;15(18):4529. doi: 10.3390/cancers15184529 (PMC10526496; doi:10.3390/cancers15184529)
Supplement: Supplementary file 1 [file cancers-15-04529-s001.zip › cancers-2335967-supplementary/cancers-2335967-supplementary.pdf]

Supplementary figures

Supplementary Figure S1

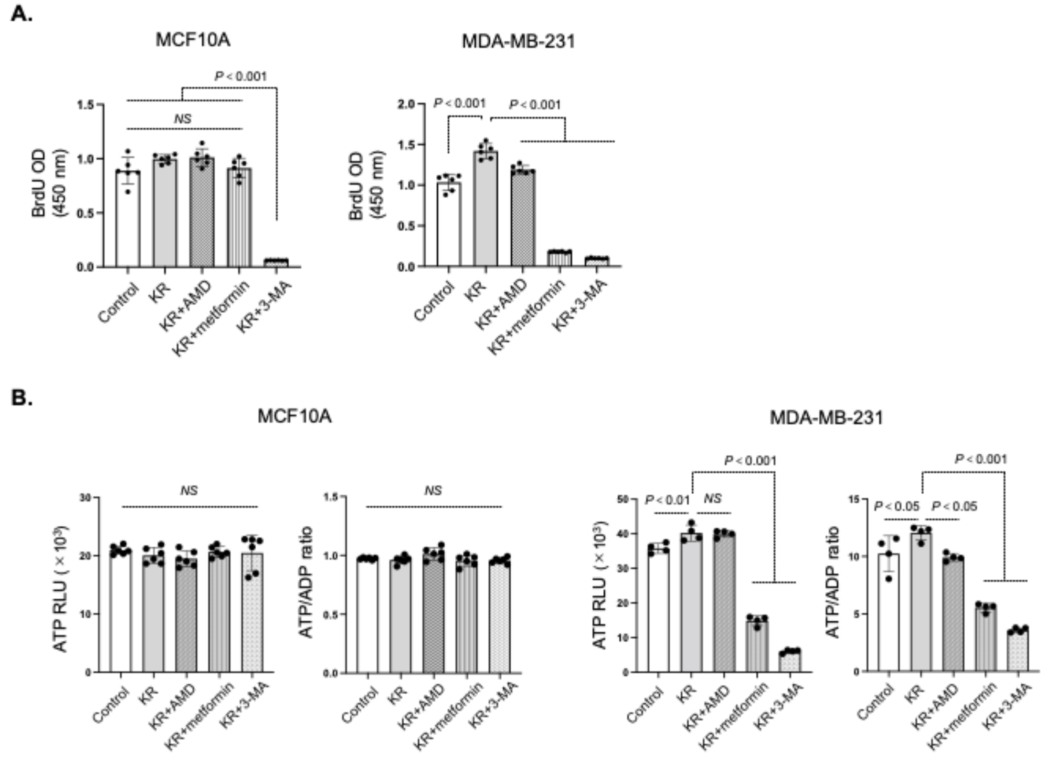

Figure S1. A. Cell proliferation was measured by BrdU incorporation assay (OD_{450}) in MCF 10A human normal epithelial cells and MDA-MB-231 human breast cancer cells after 16 h incubation with drug. The data are represented as mean ±SEM (n=6 each group). **B.** After 24 h incubation with drug, ATP and ADP levels were determined by measuring luminescence level (RLU) and ATP/ADP ratio was calculated in MCF 10A and MDA-MB-231 cells. The data in graph are presented as mean ±SEM (n=4-6 each group). **A-B.** Each cells were treated by KR62436 (KR, 50 μM) with or without AMD3100 (AMD, 30 μM), metformin (10 mM), or 3-methyladenine (3-MA, 5 mM). *NS*, not significant.

Supplementary Figure S2

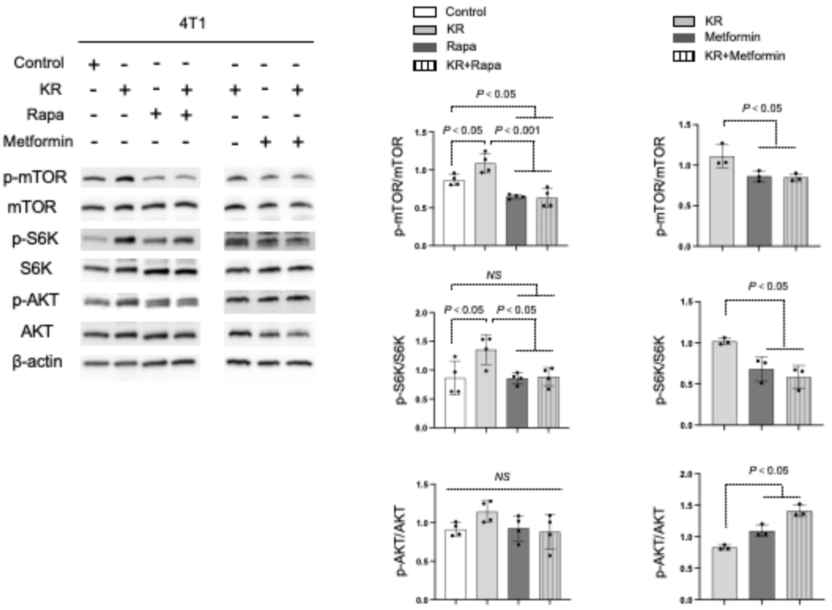

Figure S2. Western blot analysis of phosphorylated (p)-mTOR, mTOR, p-p70 S6 Kinase (S6K) (phosphorylated at Thr389), S6K, p-AKT (phosphorylated at Ser473) and AKT in 4T1 cells treated with KR62436 (KR, 50 µM), rapamycin (Rapa, 1 µM) and metformin (10 mM) for 48 h. Densitometric analysis of each protein normalized to mTOR, S6K or AKT (n=3-4 per group). The data in the graph are represented as mean ±SEM. *NS*, not significant.
