# Supplementary material for: Inhibition of Dipeptidyl Peptidase-4 Activates Autophagy to Promote Survival of Breast Cancer Cells via the mTOR/HIF-1α Pathway
Source: Cancers (Basel). 2023 Sep 12;15(18):4529. doi: 10.3390/cancers15184529 (PMC10526496; doi:10.3390/cancers15184529)

Figure1A

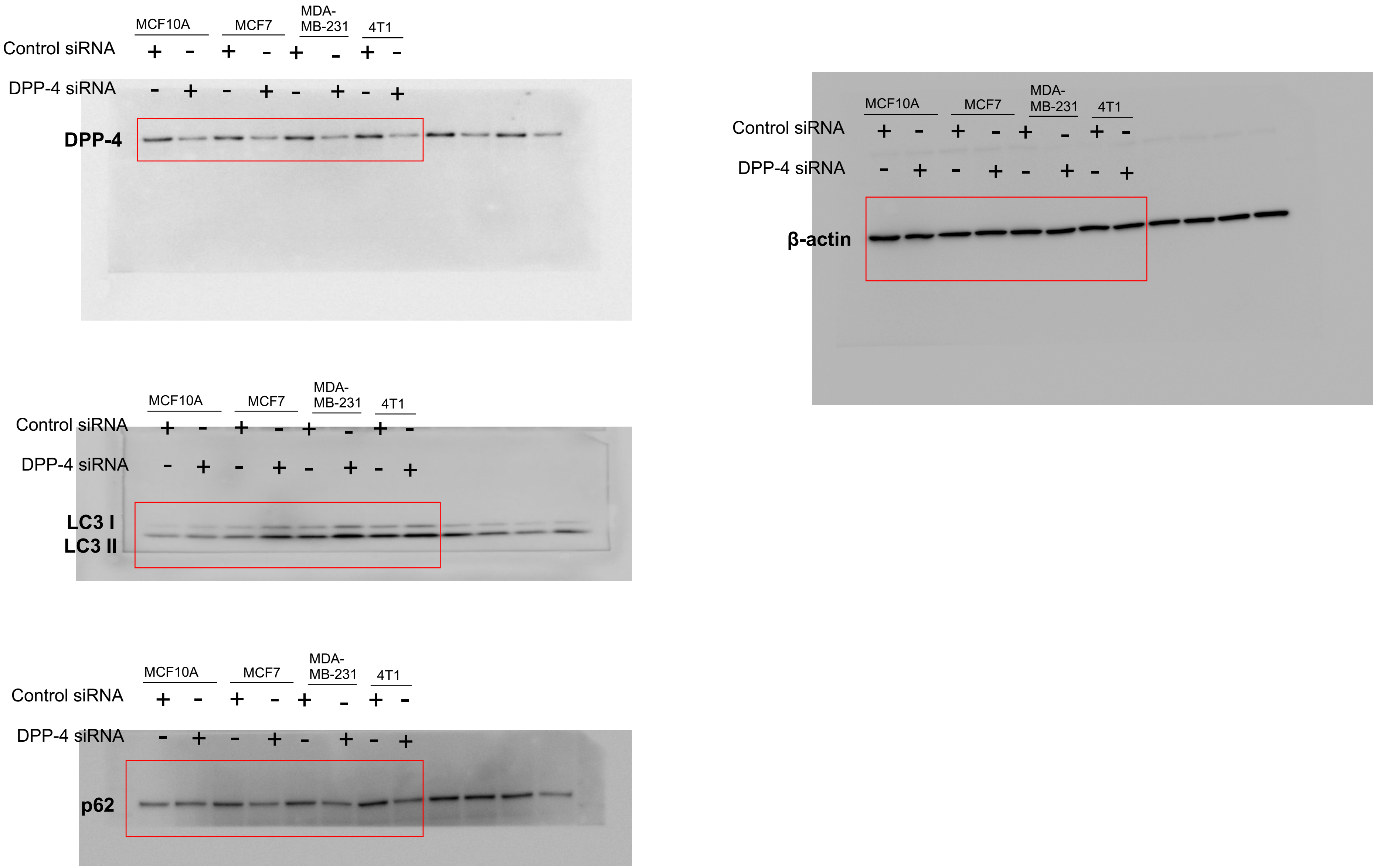

MCF10A
MCF7
MDA-MB-231
4T1
Control siRNA + - + - + - + -
DPP-4 siRNA - + - + - + - +
DPP-4
MCF10A
MCF7
MDA-MB-231
4T1
Control siRNA + - + - + - + -
DPP-4 siRNA - + - + - + - +
β-actin
MCF10A
MCF7
MDA-MB-231
4T1
Control siRNA + - + - + - + -
DPP-4 siRNA - + - + - + - +
LC3 I
LC3 II
MCF10A
MCF7
MDA-MB-231
4T1
Control siRNA + - + - + - + -
DPP-4 siRNA - + - + - + - +
p62

Figure1B

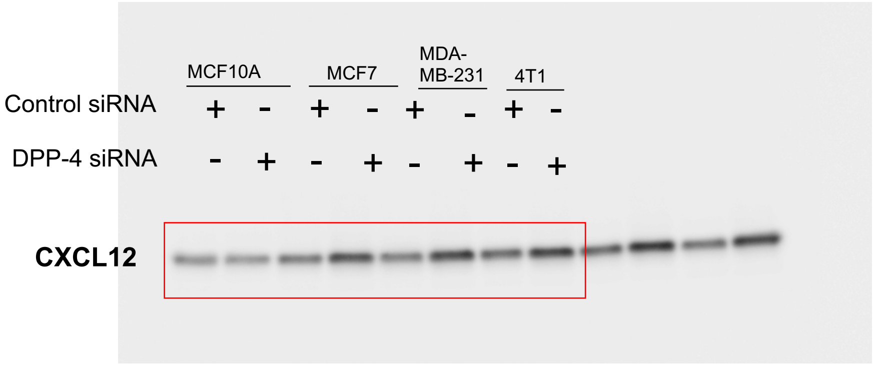
MCF10A
MCF7
MDA-MB-231
4T1
Control siRNA + - + - + - + -
DPP-4 siRNA - + - + - + - +
CXCL12

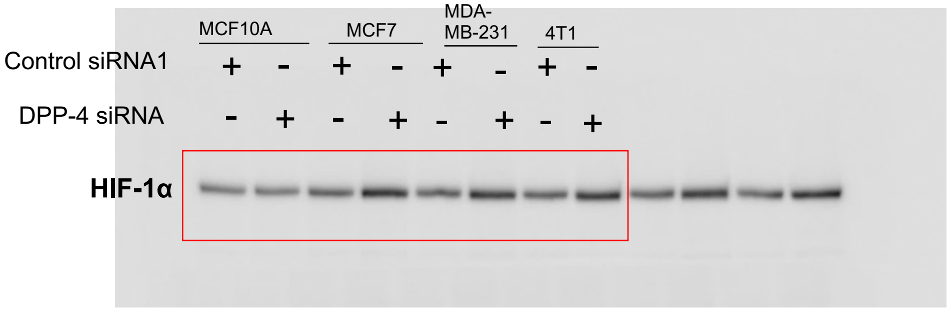
MCF10A
MCF7
MDA-MB-231
4T1
Control siRNA1 + - + - + - + -
DPP-4 siRNA - + - + - + - +
HIF-1α

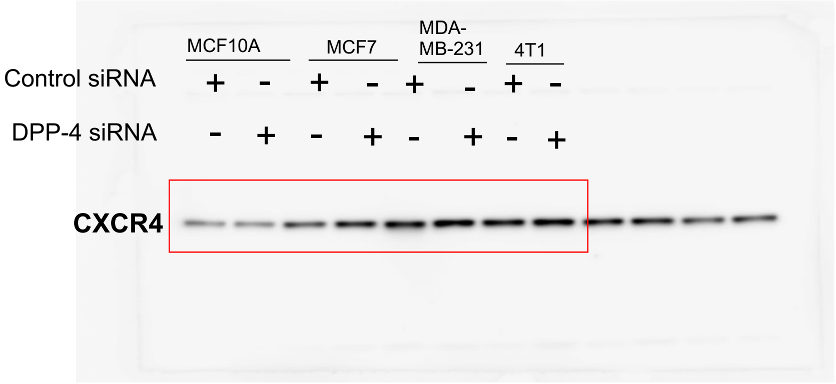
MCF10A
MCF7
MDA-MB-231
4T1
Control siRNA + - + - + - + -
DPP-4 siRNA - + - + - + - +
CXCR4

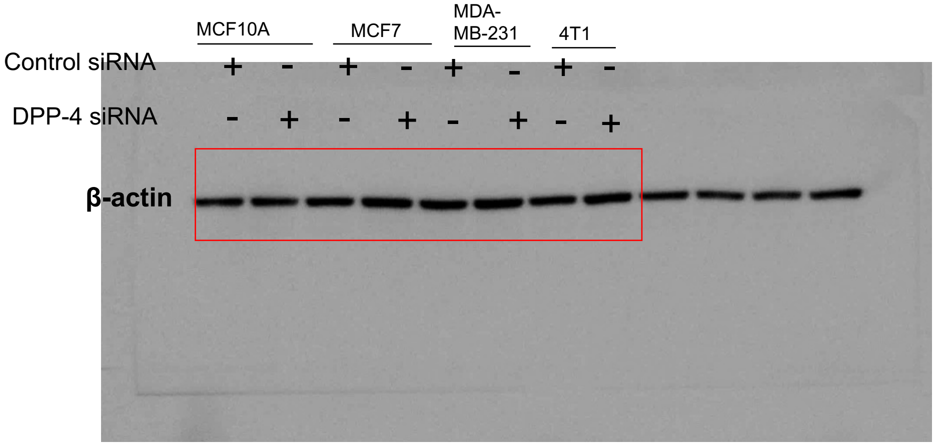
MCF10A
MCF7
MDA-MB-231
4T1
Control siRNA + - + - + - + -
DPP-4 siRNA - + - + - + - +
β-actin

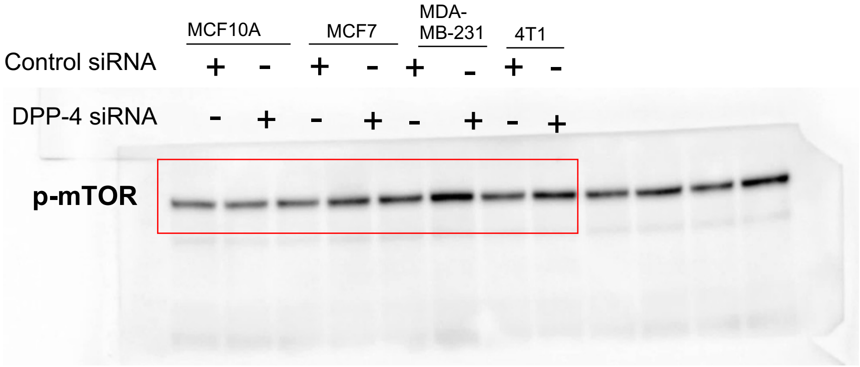
MCF10A
MCF7
MDA-MB-231
4T1
Control siRNA + - + - + - + -
DPP-4 siRNA - + - + - + - +
p-mTOR

Figure2A

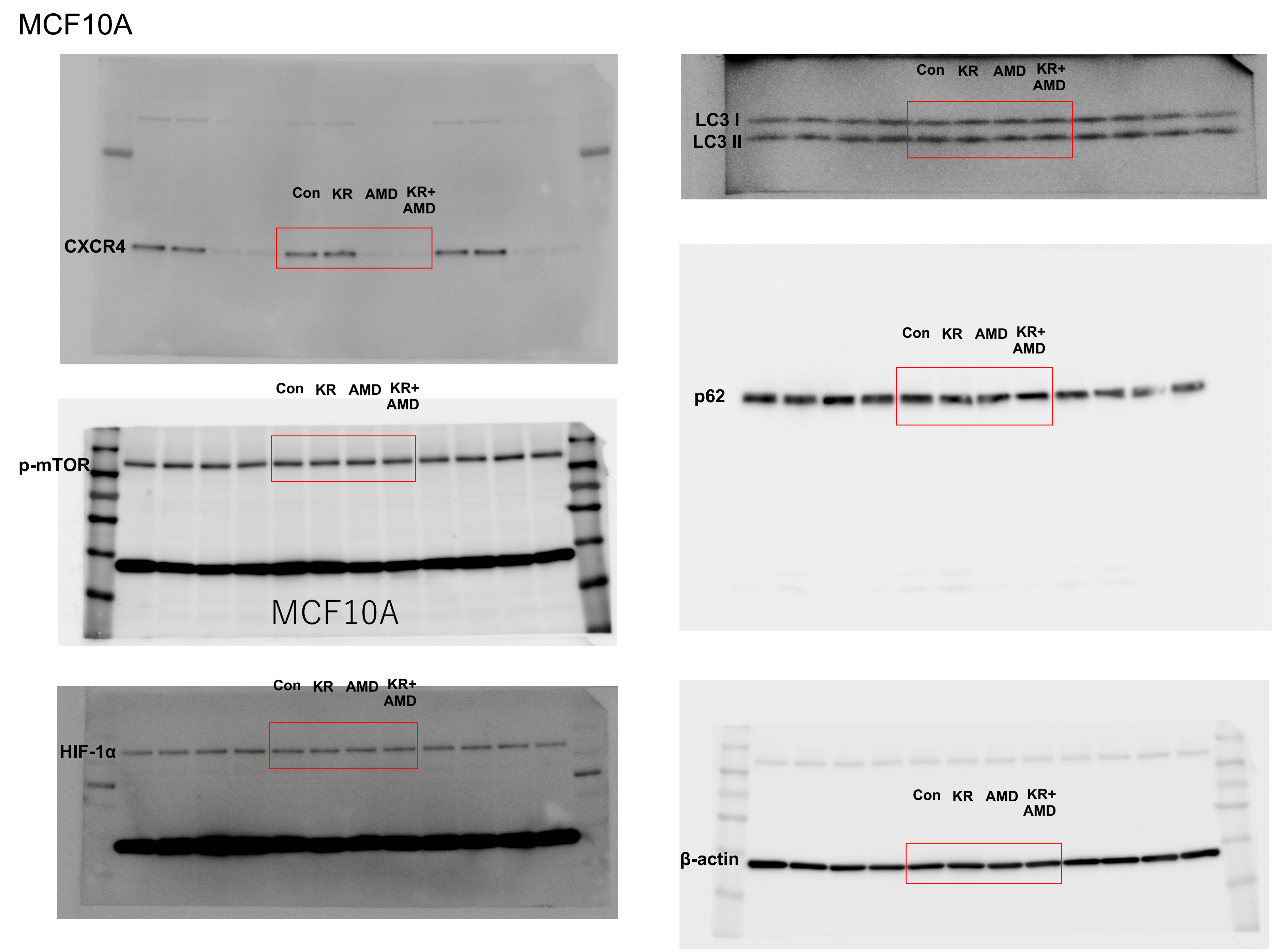
MCF10A
Con KR AMD KR+ AMD
CXCR4
Con KR AMD KR+ AMD
p-mTOR
MCF10A
Con KR AMD KR+ AMD
HIF-1α
Con KR AMD KR+ AMD
LC3 I
LC3 II
Con KR AMD KR+ AMD
p62
Con KR AMD KR+ AMD
β-actin

Figure2A MCF7

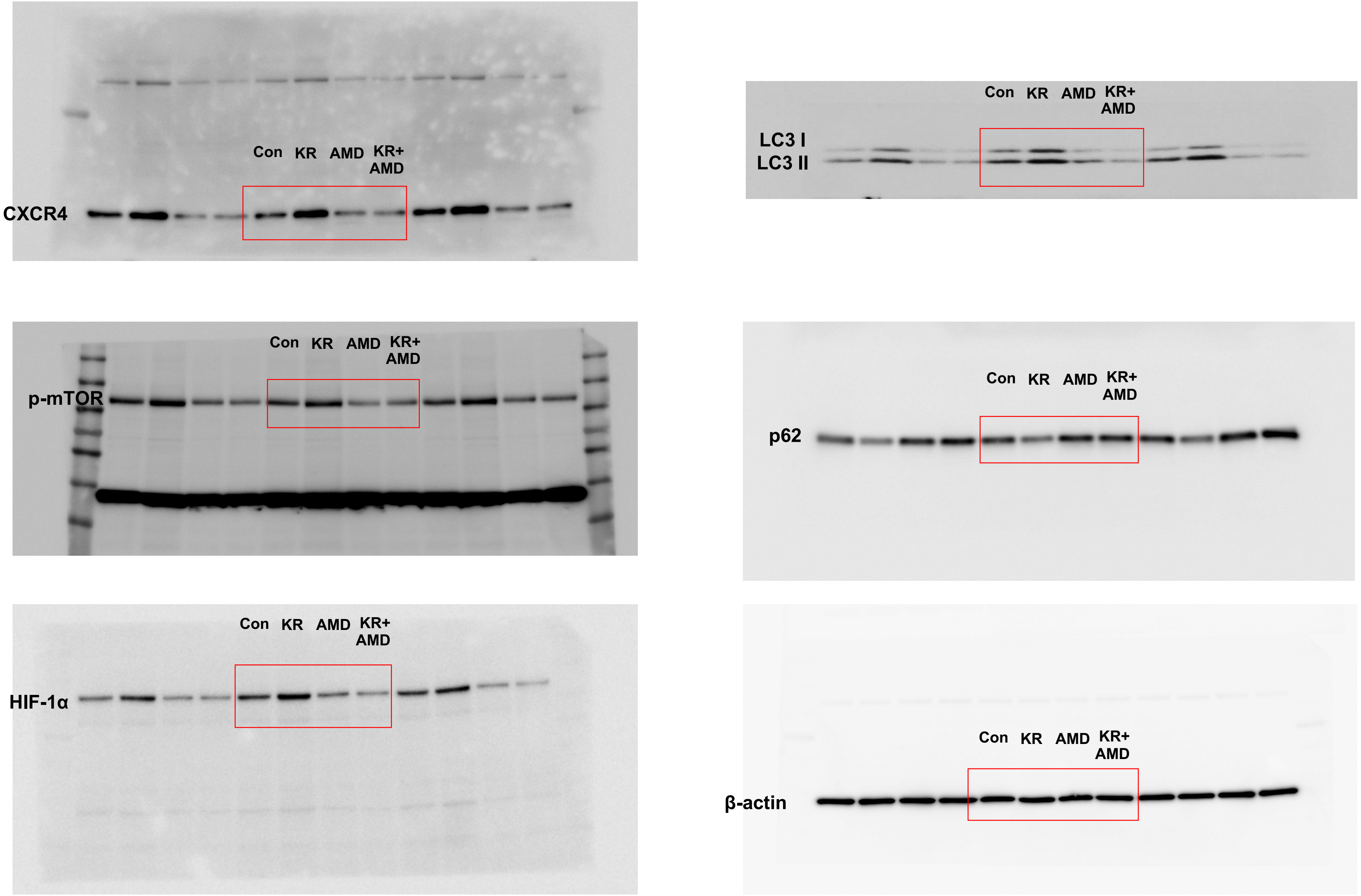

Figure2A

MDA-MB-231

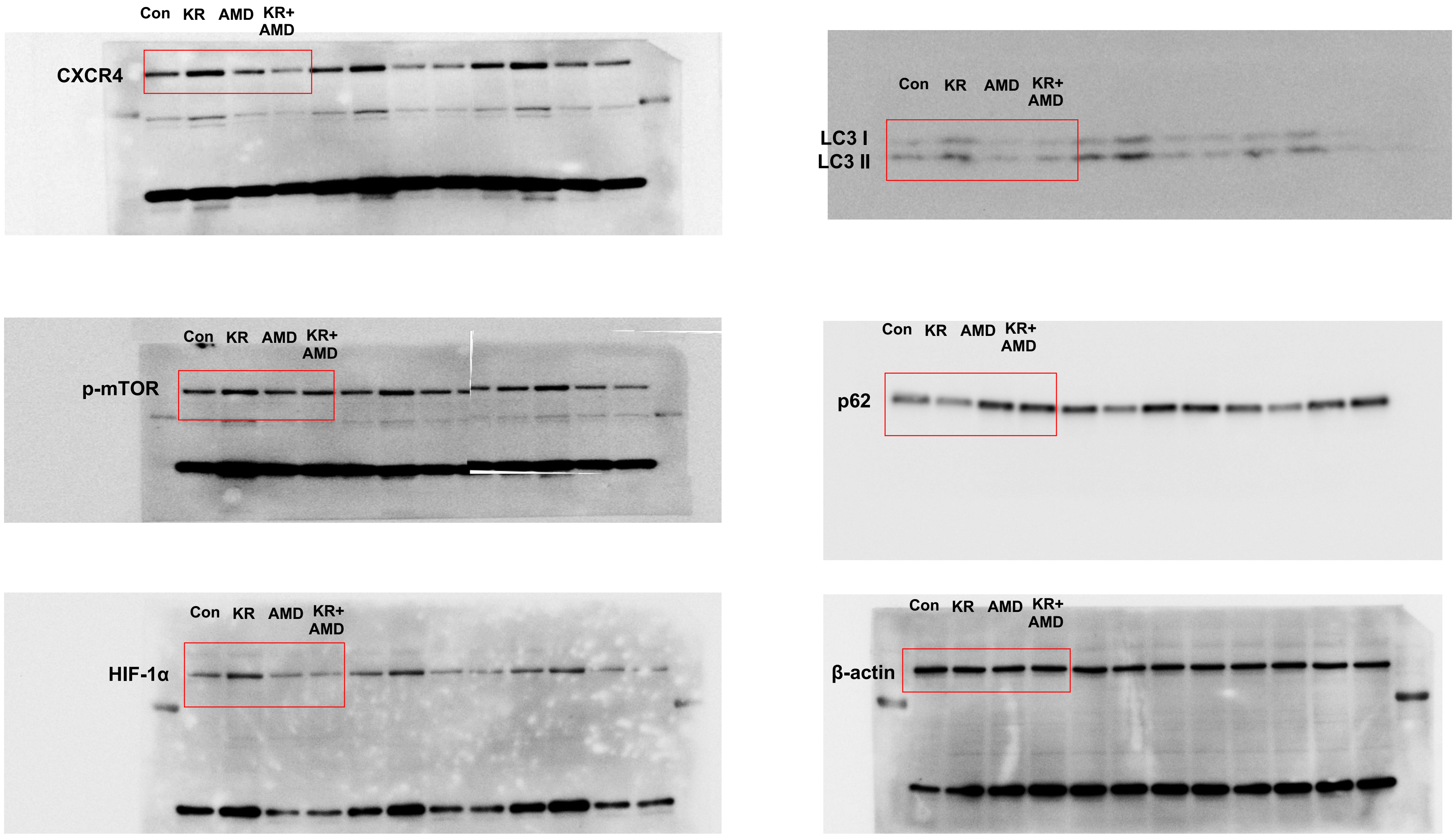

Figure2B

4T1

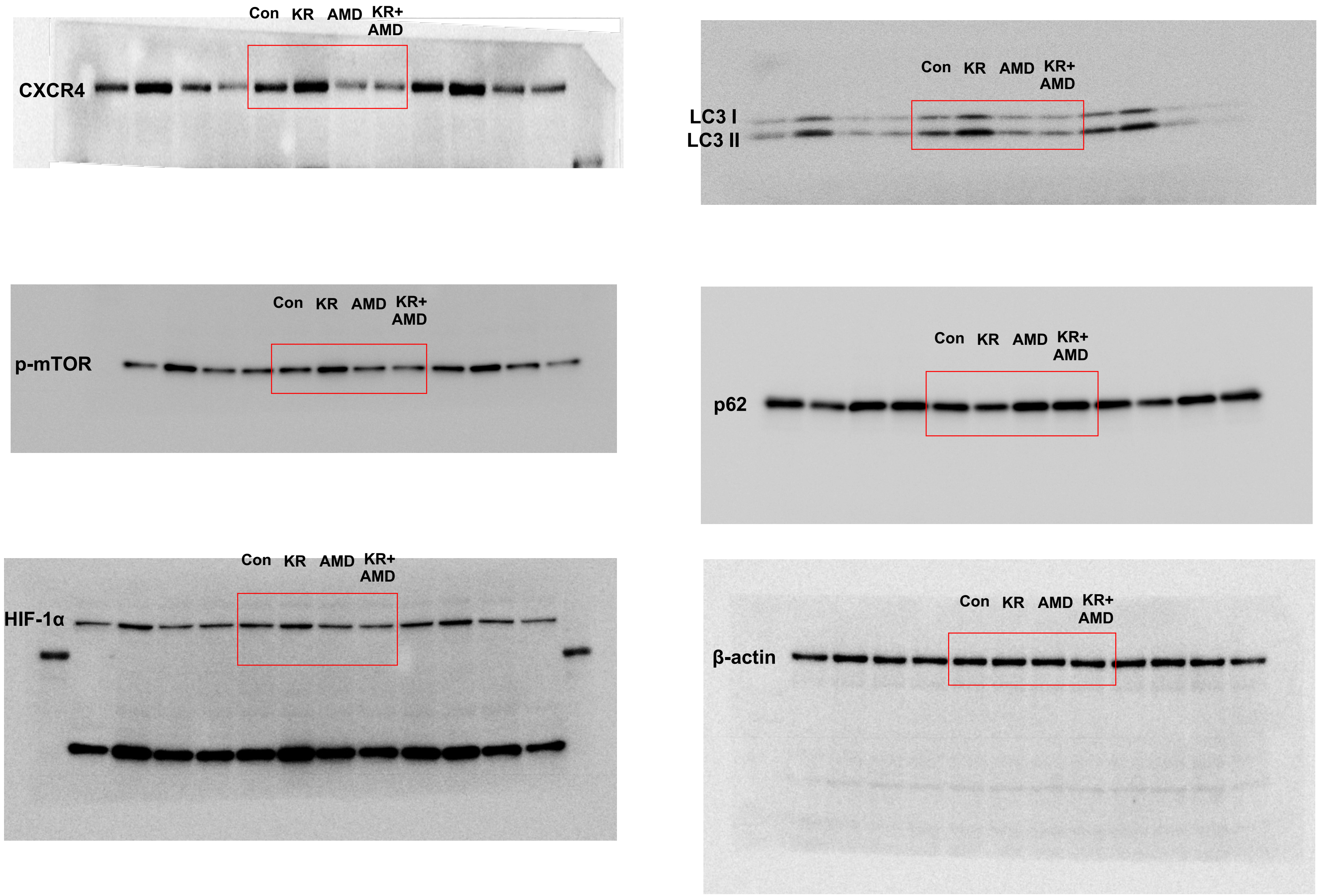

Figure3A

MCF10A

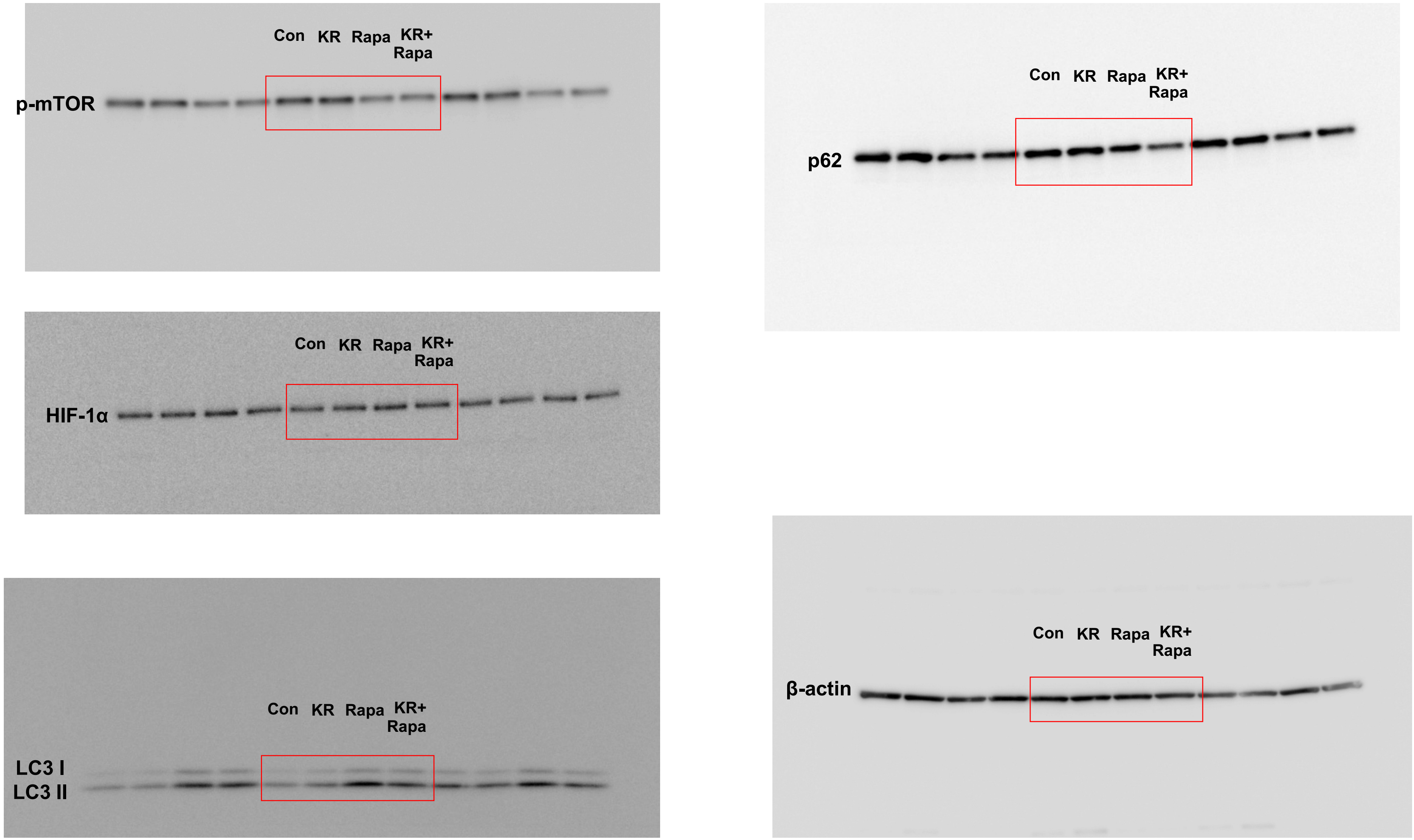

Figure3A MCF7

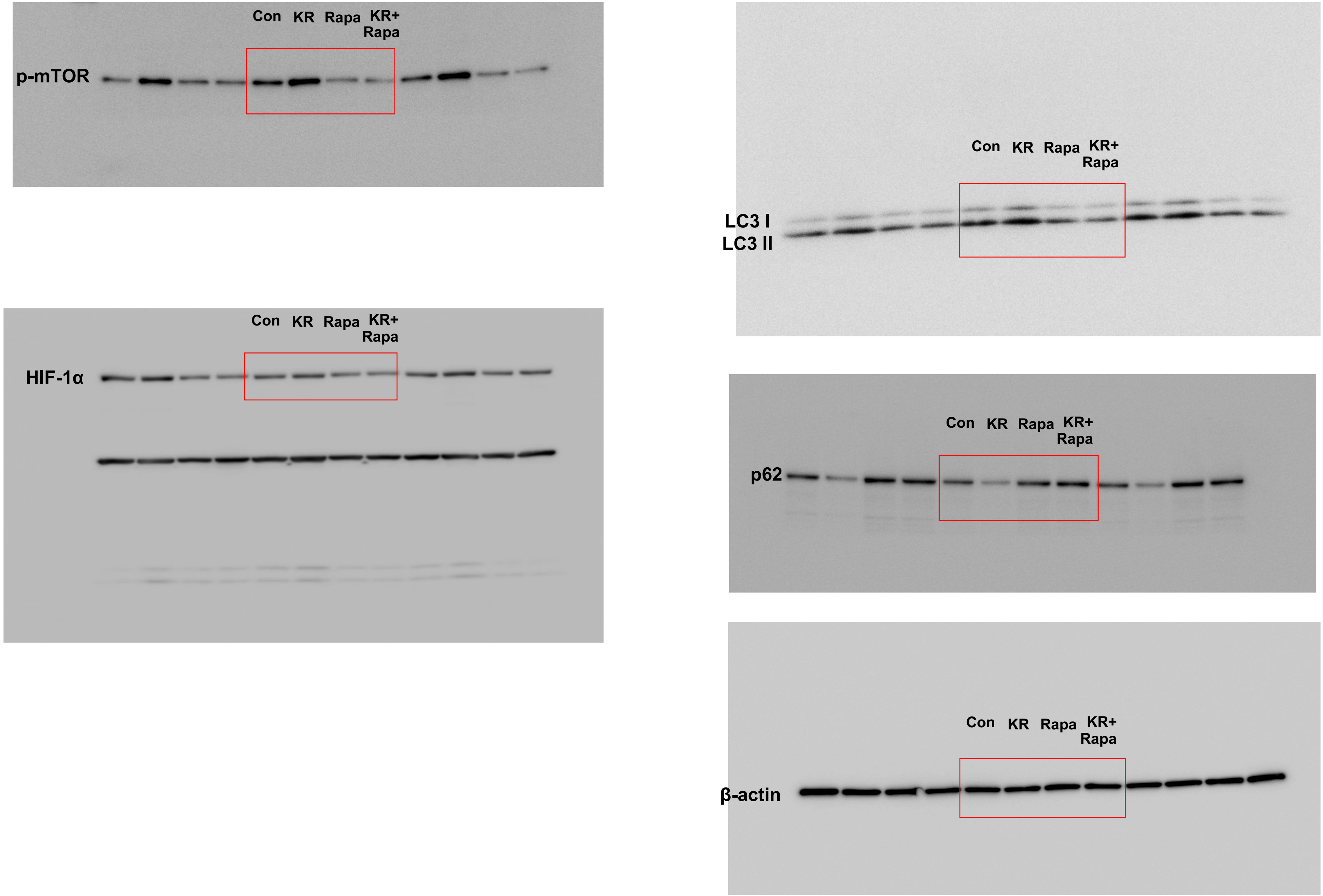

Figure3A

MDA-MB-231

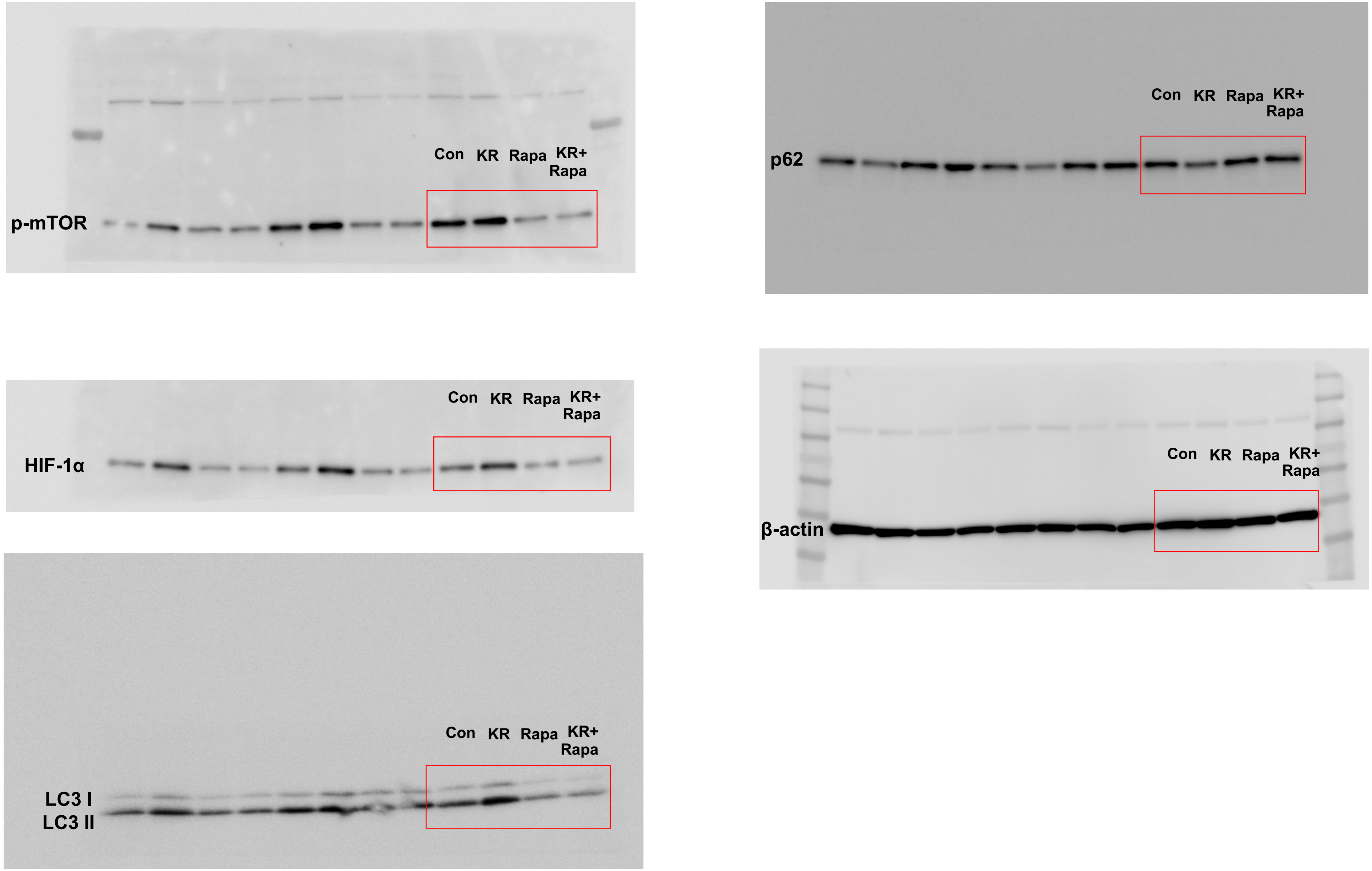

Figure3B

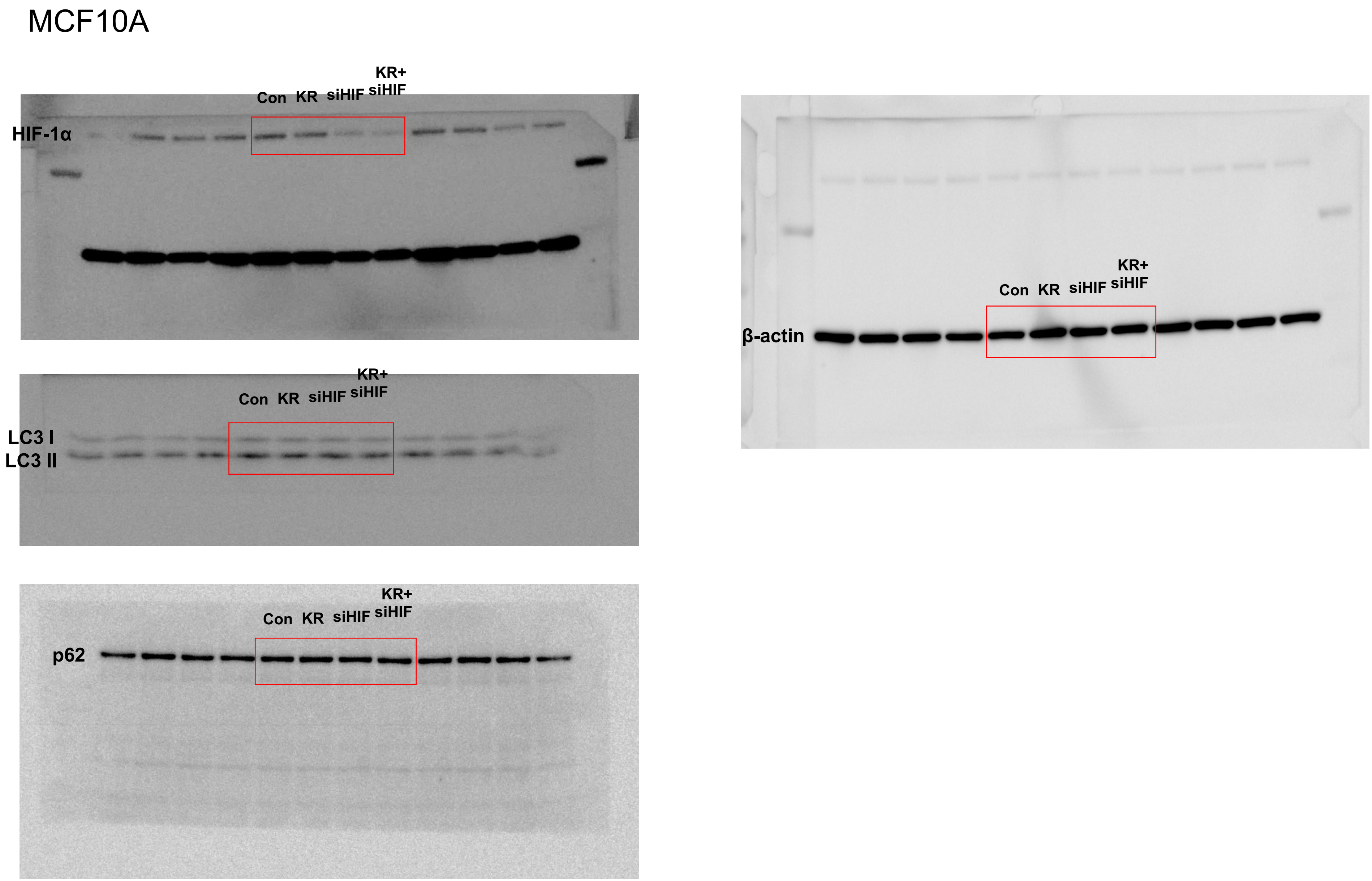

Figure3B

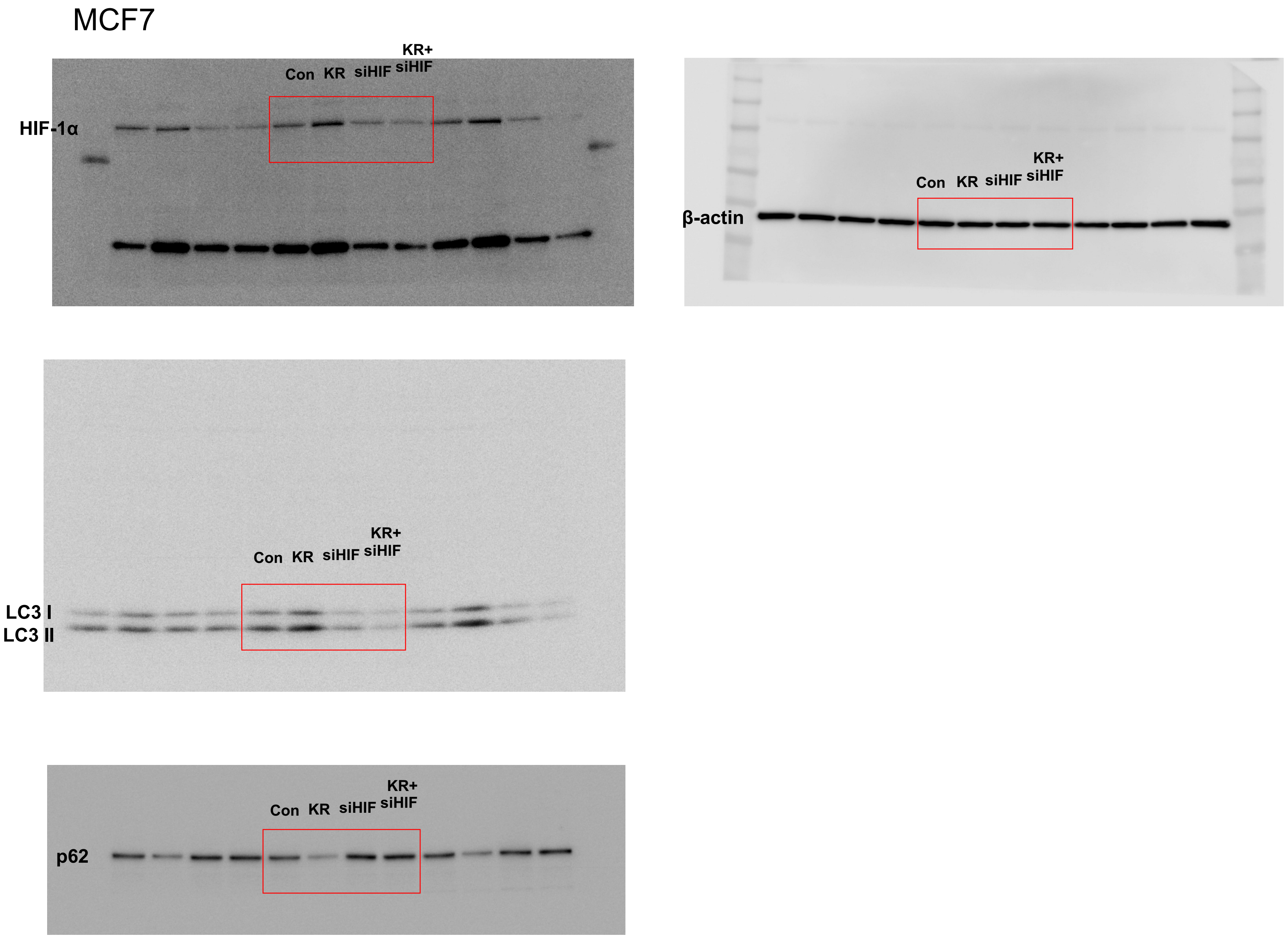

Figure3B

MDA-MB-231

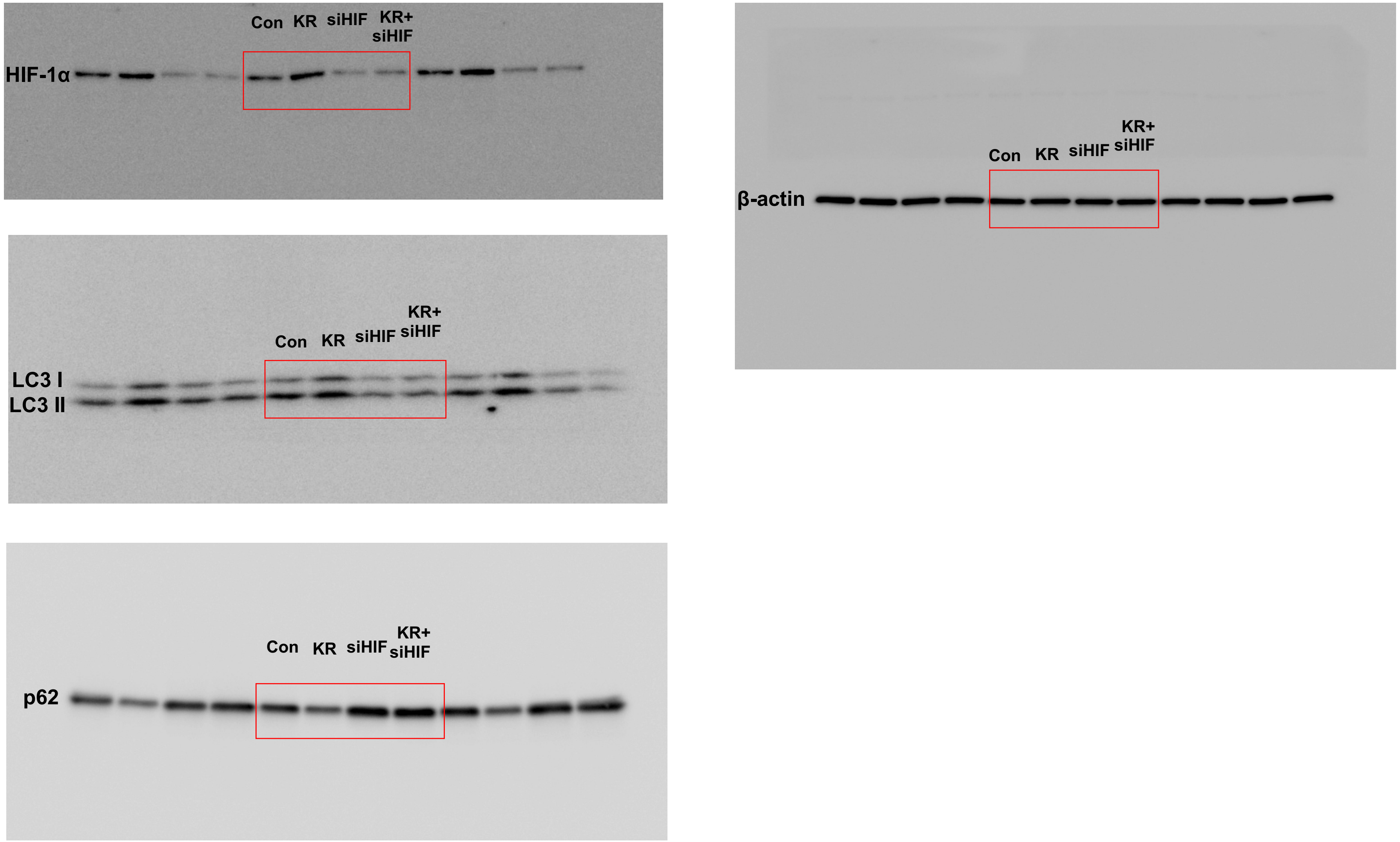

Figure 4A

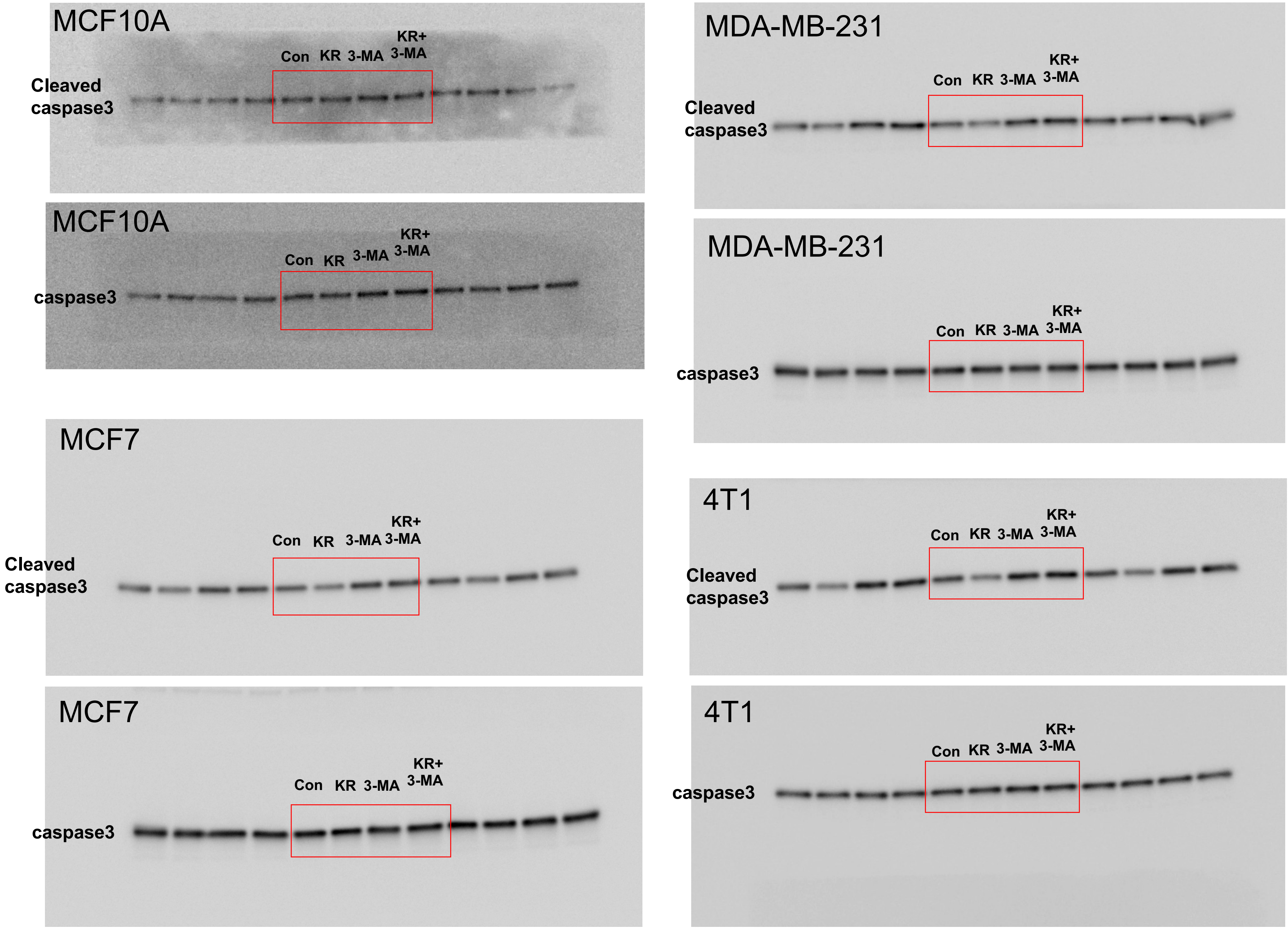
MCF10A
Con KR 3-MA KR+ 3-MA
Cleaved caspase3
MCF10A
Con KR 3-MA KR+ 3-MA
caspase3
MCF7
Con KR 3-MA KR+ 3-MA
Cleaved caspase3
MCF7
Con KR 3-MA KR+ 3-MA
caspase3
MDA-MB-231
Con KR 3-MA KR+ 3-MA
Cleaved caspase3
MDA-MB-231
Con KR 3-MA KR+ 3-MA
caspase3
4T1
Con KR 3-MA KR+ 3-MA
Cleaved caspase3
4T1
Con KR 3-MA KR+ 3-MA
caspase3

Figure 5A

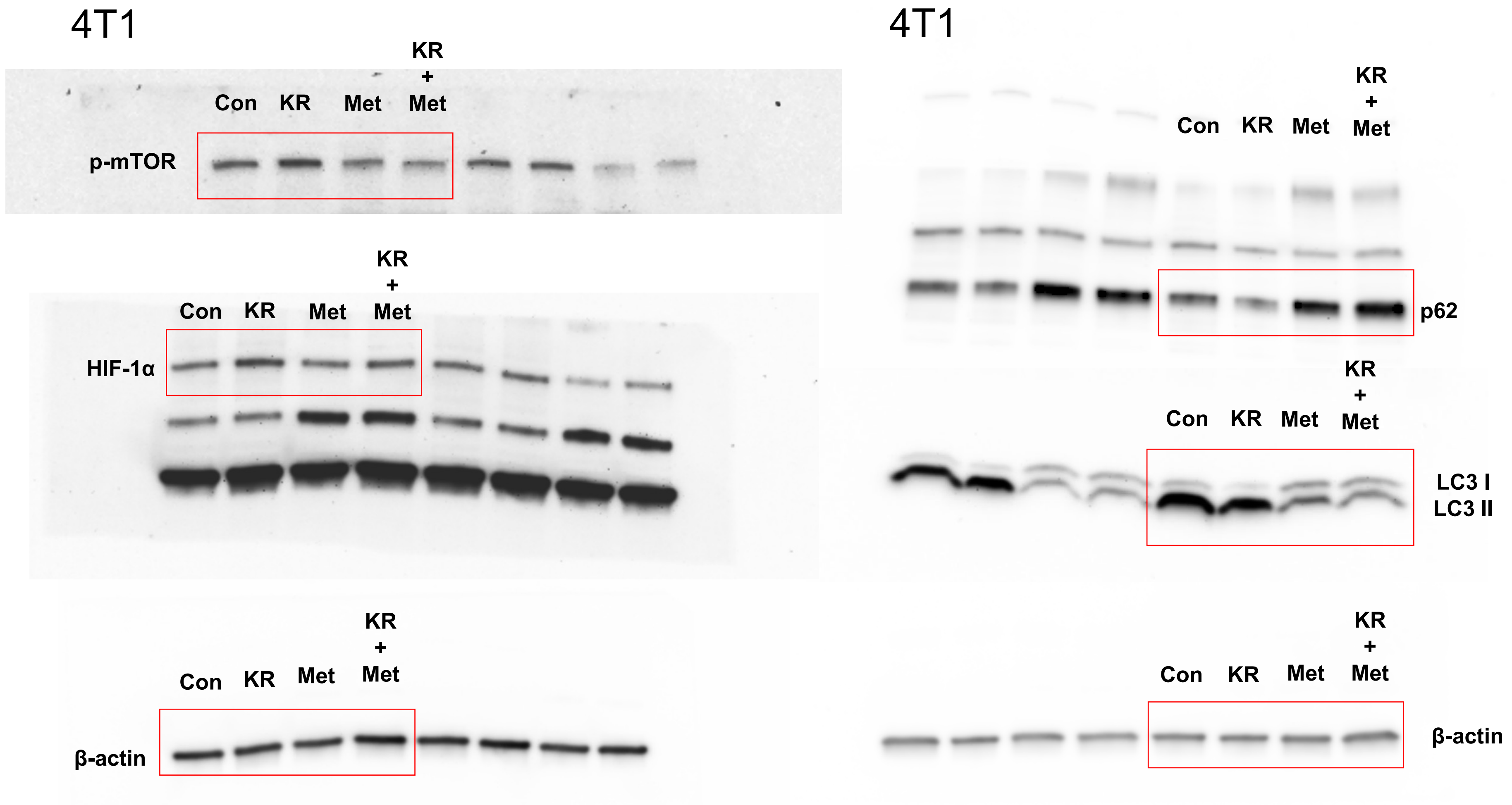
4T1
Con
KR
Met
KR
+
Met
p-mTOR
HIF-1α
β-actin
4T1
p62
LC3 I
LC3 II
β-actin

Supplementary Figure S2

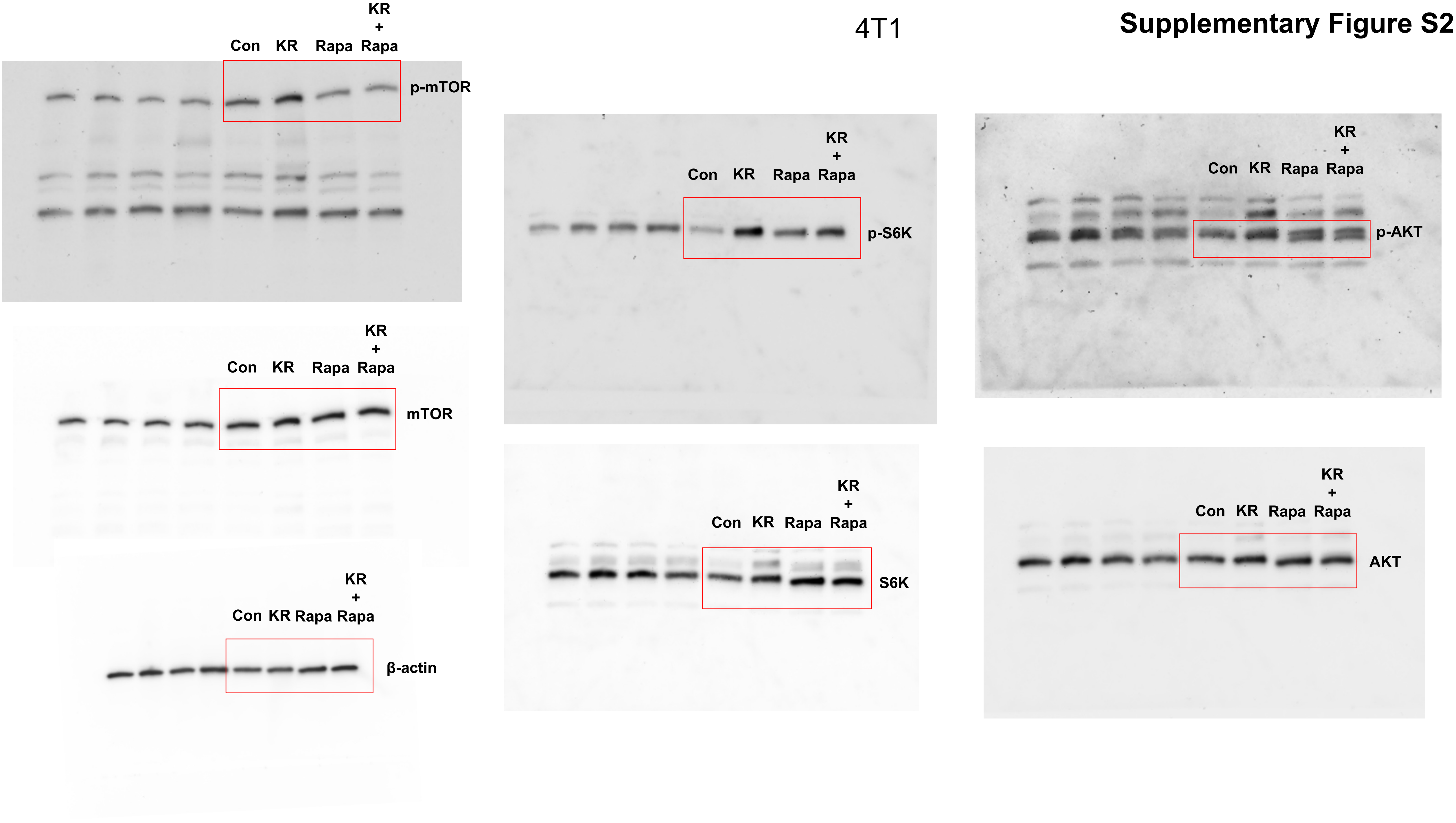

Supplementary Figure S2

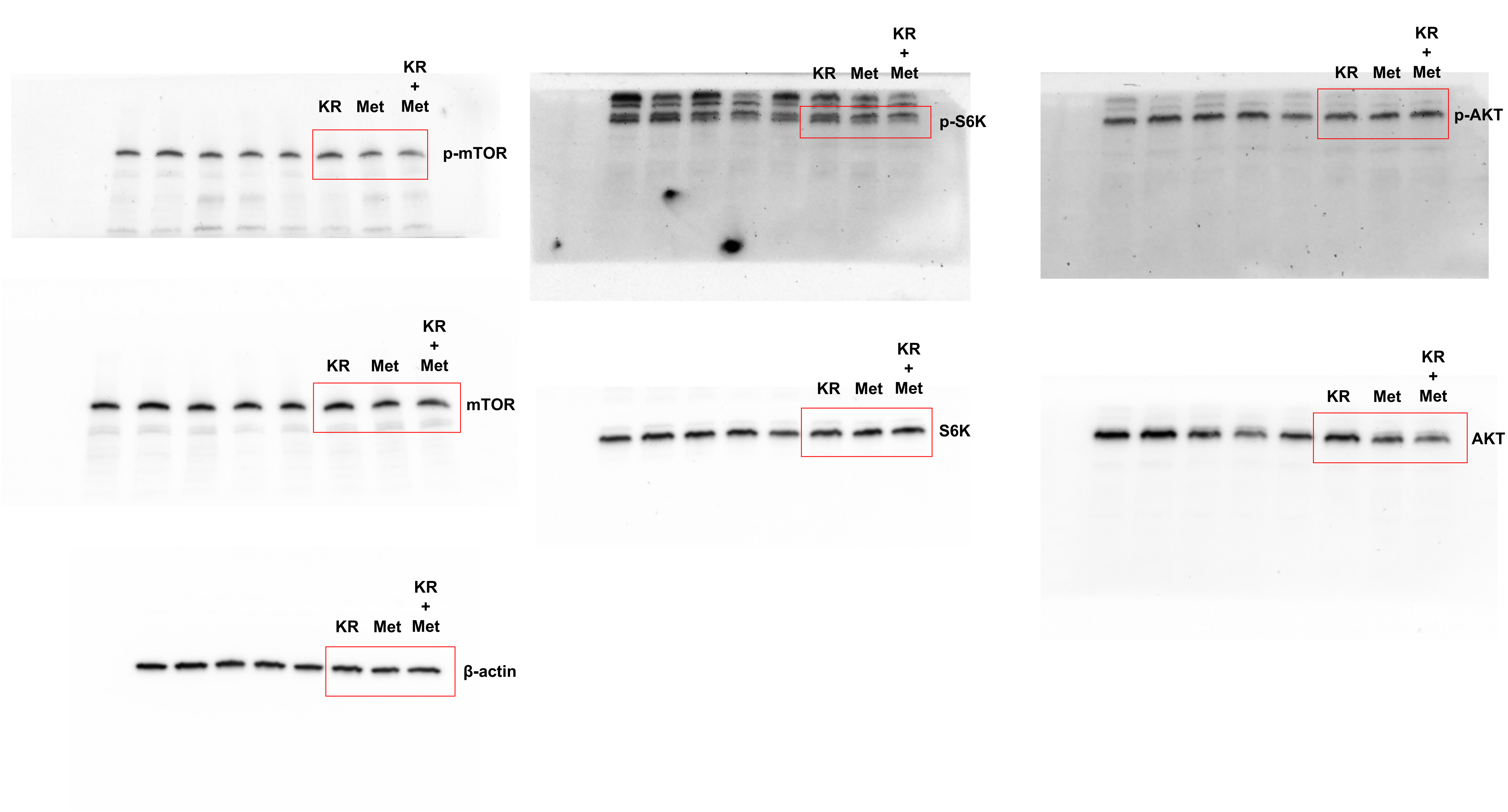

Supplement: Supplementary file 1 [file cancers-15-04529-s001.zip › cancers-2335967-supplementary/Western blots.pdf]
